# Gaps in Transgender Medicine Content Identified Among Canadian Medical School Curricula

**DOI:** 10.1089/trgh.2016.0010

**Published:** 2016-07-01

**Authors:** Benjamin Chan, Rachel Skocylas, Joshua D. Safer

**Affiliations:** ^1^ Faculty of Medicine, Department of Undergraduate Medicine, University of British Columbia, Vancouver, Canada.; ^2^ Department of Endocrinology, Diabetes, and Nutrition, Boston University School of Medicine, Boston, Massachusetts.

**Keywords:** clinical care, health education/training programs, survey design or survey methodology, transgender

## Abstract

**Purpose:** The transgender community is a diverse group that requires unique consideration in the healthcare setting. However, several studies have suggested that their needs are not currently being met by our medical system. Although the reason for this discrepancy is likely multifactorial, inadequate training of healthcare professionals to manage this population has been cited as a contributing factor.

**Methods:** To evaluate the role that Canadian medical schools play in addressing these proposed deficits, program administrators were invited to provide curricular information detailing their delivery of transgender health, and medical students were surveyed to assess the impact of current curricula on their knowledge, attitudes, and experiences with regard to transgender health.

**Results:** Six of fourteen schools provided curricular information about their instruction in transgender health and wide variation was found; 255/1152 University of British Columbia (UBC) students and 155/2358 students from eight other Canadian medical schools responded to the survey. Greater than 95% of responders agreed that transgender issues are important and should be addressed by physicians. However, fewer than 10% of students felt that they were sufficiently knowledgeable to do so. At UBC, there was no significant improvement in the self-reported knowledge levels after receiving the transgender-related curricula, and only 24% of students felt the topic was proficiently taught.

**Conclusion:** This study showed that the majority of students who responded do not feel comfortable addressing the needs of transgender individuals in a healthcare setting and suggests that a reevaluation of related curricula may be warranted.

## Introduction

The transgender community is a diverse group that represents a sizeable proportion of the population.^1–4^ It is not only made up of individuals wishing to transition, but it is also a spectrum that includes individuals who simply hold an identity that is not associated with their sexual anatomy.^3,4^ Mayer et al. defined the community as one that is made up of individuals who have gender identities, expressions, or behaviors not traditionally associated with their natal sex.^5^^(p. 990)^ Due to this broad definition, the exact size of the group is difficult to measure^6,7^ and many health registries lack any statistics. Nevertheless, in 2011, the Institute of Medicine suggested that the transgender community may represent 0.3–0.5% of the adult population^8^; however, many have argued that even this may be an underestimation.^4^

Transgender individuals also have a unique set of needs within the healthcare setting.^9–11^ Most notably, the population requires particular sensitivity. Care needs to be given by the healthcare team in a nondiscriminatory manner to ensure gender identities of the individuals are respected.^12–14^ For example, healthcare providers need to be conscious of the individuals' preferred pronoun.^9^ Providers must also be wary of becoming overly focused on the gender identity of their transgender patients and neglecting their general healthcare needs.^15,16^ For example, even though one may identify as a male, if one has breast tissue, one must still receive regular mammograms as per the appropriate guidelines.^15^ Furthermore, healthcare professionals should also be aware of the options available to transgender individuals wishing to transition, that is, medical and surgical interventions, so that they can either provide patients with this knowledge themselves or know when to refer these patients.^3,13^

Despite their prevalence in the community and their unique needs, transgender individuals continue to be underserved.^9,10,13,17^ For example, transgender individuals do not receive adequate cancer screenings.^15–17^ There continues to be a large disparity in the number of transgender men receiving regular mammograms in comparison with cisgendered females.^15^ Similar disparities are seen for cervical cancer screenings.^17^ Additionally, disrespect and discrimination are still problematic in many physicians' offices.^4,11,16^ This has not only shown to impact the likelihood that transgender individuals seek medical care, but also may play a role in the poorer reported health of the community in comparison with the general population.^18,19^

Many factors may be contributing to the poorer health and healthcare of the transgender community. These may include barriers at an individual level, such as personal prejudices,^3,4,20^ as well as at an institutional level, such as those involving political legislations and healthcare delivery.^9,21,22^ However, even in societies with advanced policies designed to protect transgender individuals, the community continues to be underserved.^9,21^ In Canada, there have been major legislative developments to better the treatment of transgender individuals, such as explicit human rights laws protecting gender identity and expression.^9,23^ Furthermore, the publicly funded medical service plan, designed to make essential healthcare services equally available to all citizens, has made many transgender-related services such as gender-confirming surgery publicly available.^9,23^ However, despite these developments, the needs of transgender individuals are not being met.^18,19^

Many have suggested that inadequate training in healthcare may be contributing to the poorer health outcomes in this population^14,20,24^ as gaps in transgender health have been shown to exist in the training of healthcare professionals, including physicians and nurses, worldwide.^21,22,24^ To investigate the role that the training of physicians in Canada may play in the delivery of healthcare to transgender individuals, we chose to evaluate the extent of coverage of transgender health in medical schools across Canada and assess student's comfort level regarding the topic. We focused our study on medical students at the University of British Columbia (UBC) and compared them with students at other Canadian medical institutes.

## Methods

### Study design

The study had three objectives. The first was acquisition of curricular data. The second was a cross-sectional study of medical students at the UBC. The third was a cross-sectional study of medical students throughout the rest of Canada.

### Study protocol

#### Curriculum acquisition

Program administrators who were able to comment on the instruction of transgender health at their institute from medical schools across Canada were invited to provide curricular data regarding the instruction of transgender health. An email was sent to the undergraduate office of the institute, asking to be directed to a program administrator who would best be able to address questions concerning transgender-related curricular content. Those administrators were then provided with a short standardized survey that consisted of five questions, addressing how many hours are spent on the topic, when the topic is introduced in the curricula, how the information is delivered, and what material is covered. Administrators were also asked to select which of the following topics were covered at any point in their curricula: gender dysphoria, transgender-specific risks to address in history taking, transgender-specific risks to address in physical exams, hormone therapy, and transgender surgery. Administrators were not asked to differentiate between material presented in the general curriculum and material available in electives.

#### Student survey

Medical students in all years were surveyed at UBC as well as other schools across Canada. The survey consisted of 26 multiple choice questions and was constructed using *Fluid Surveys*. Surveys from previously published works were used as a reference.^11,17,19^ Consent was obtained from the authors to use those surveys.

Ethics approval for the study was obtained from the Behavioral Research Ethics Board (BREB) at UBC as well as the Research Access Committee (RAC) at UBC.

There were two identifier questions (which asked the students to report the school they attend and their year of study), six questions assessing students' experiences with the transgender community, three questions assessing students' experiences with transgender medicine in their curricula, six questions assessing students' attitudes toward transgender health, and nine questions assessing students' knowledge of transgender health. The majority of questions asked students to evaluate whether they strongly agreed, agreed, did not know, disagreed, or strongly disagreed with a statement pertaining to their attitudes, experience, or knowledge with regard to transgender health.

Knowledge-based questions asked students to evaluate whether a statement relating to transgender health was true or false. These questions were designed to touch on various topics within transgender health and prompt students to think more deeply about how much or how little they knew about the topic before reporting on their comfort level with regard to their knowledge of transgender health.

### Sample size

All 1152 medical students currently at UBC—in all years—were invited to complete the survey. Two thousand three hundred fifty-eight students from other Canadian medical schools were invited to complete the survey. This pool represented all students from the participating schools that were reachable with a standard listserv interface. All 13 of the other Canadian medical schools were invited to participate, and the following eight schools participated: University of Alberta, University of Calgary, University of Saskatchewan, University of Manitoba, University of Toronto, Western University, Northern Ontario School of Medicine, and Memorial University. Survey responses from past students (i.e., current residents) were excluded.

### Sampling methods

Program administrators were invited to voluntarily complete a short survey through email. The email was sent to the undergraduate office and then directed to the appropriate program administrator. If the undergraduate office did not respond, a follow-up email was sent.

Medical students were invited to voluntarily complete the survey through email. The email was sent through a medical school-wide email listserv. Consent was obtained in the survey—students were not able to complete the survey unless they provided consent. To encourage students to respond, students were given an opportunity to enter into a draw to win a 1 of 20 ($5) *Starbucks* gift card.

### Data analysis

Survey responses were exported from *Fluid Surveys* and analyzed using *Microsoft Excel*. Three questions were excluded from the analysis because they were found to be out of scope, repetitive, or to have ineffective wording. Every question may not have been answered in every student survey response as students did not have to answer every question to submit the survey. For comparison purposes, the strongly agree and strongly disagree responses were grouped together with the agree and disagree responses, respectively. Unpaired *t* tests were conducted on select questions to determine whether or not a significant difference existed among different groups (i.e., first year vs. years 2–4, and UBC students vs. students at other Canadian medical schools) with *p* values less than 0.05 set as a significant difference.

## Results

Six schools provided curricular information about their instruction in transgender health (Table 1). Large differences were found in the time spent, ranging from 0 to 2 h to greater than 8 h. Differences were also seen in the way students were introduced to the topic. Some schools taught the material in discrete sections, while other schools dispersed transgender-related teachings throughout the medical curricula. Furthermore, some schools reported covering all the transgender-related topics listed in the Methods section, while other schools reported covering a selection. Every school that provided data reported covering the topic of gender dysphoria.

**Table 1.** **Variability in the Hours Spent, Method of Instruction, Point of Introduction, and Specific Topics Covered with Regard to Transgender-Related Curricula in Canadian Medical Schools**

	Curricular qualities			Topics covered				
School	How many hours spent?	How is the topic taught?	When is the topic covered?	Gender dysphoria	Transgender considerations in the physical examination	Transgender considerations in a history	Hormone replacement therapy	Gender-affirming surgeries
University of British Columbia	2–4	Discrete sections	End of first year	x	x	x		
University of Saskatchewan	>8	Throughout curricula	End of second year	x	x	x	x	x
University of McGill	0–2	Discrete sections	End of second year	x	x	x		x
University of Alberta	>8	Throughout curricula	End of fourth year	x	x	x	x	x
University of Calgary	2–4	Discrete sections	End of second year	nd	nd	nd	nd	nd
Memorial University	0–2	Discrete sections	End of first year	nd	nd	nd	nd	nd

nd, no data; x, topic covered.

The survey results from students at UBC as well as eight other Canadian medical institutes from each question were compiled (Tables 2–5). The response rate was 22.1% for UBC and 6.7% for the other Canadian institutes. At UBC, of the 1152 medical students, 255 responded. Of those students, 79 were in first year, 110 were in second year, 46 were in third year, and 20 were in fourth year. Of 2358 students surveyed from the other Canadian medical schools, 155 responded. Of those students, 25 were in first year, 54 were in second year, 32 were in third year, and 41 were in fourth year.

**Table 2.** **UBC and Canadian Medical Students' Reported Attitudes Toward the Transgender Community in General and the Transgender-Related Curricula at Their Respective Institute**

	UBC (*N*=255)			Canada (*N*=110)		
Question	% Agree or strongly agree	% Disagree or strongly disagree	% Do not know	% Agree or strongly agree	% Disagree or strongly disagree	% Do not know
1. I feel comfortable around transgender individuals.	82	10	8	92	4	4
2. My upbringing has negatively affected the way I perceive transgender individuals.	16	80	4	6	88	6
3. My experiences with transgender individuals have positively affected my thoughts about what it means to be transgender (if you have not met a transgender individual, select “I do not know”).	61	5	35	71	5	24
4. Transgender individuals have unique health risks.	92	1	7	95	3	2
5. I feel that transgender health is best treated as a psychological condition.	7	74	19	8	78	14
6. As a physician, I feel it is important for me to know if my patients are struggling with their gender identity.	99	0	1	98	1	1
7. I believe that being transgender is wrong.	1	93	6	1	98	1
8. I was introduced to transgender health in my medical curricula.^a^	72	26	2	67	31	2
9. I felt the topic of transgender health was proficiently taught.^a^	24	69	7	27	70	3
10. I was introduced to a transgender person (i.e., colleague, professor, patient) in my medical education.^a^	44	53	3	52	43	5
11. I would like to know more about transgender health.	88	6	6	88	6	6

^a^ Responses from first-year students were excluded from the results shown for UBC for these questions as students had not yet received the transgender-related curricula.

UBC, University of British Columbia.

**Table 3.** **UBC and Canadian Medical Students' Reported Experience with Transgender Individuals in the Community**

	UBC (*N*=255)			Canada (*N*=110)		
Question	Yes (%)	No (%)	I do not know (%)	Yes (%)	No (%)	I do not know (%)
12. I identify as transgender.	0.5	99	0.5	0	99	1
13. I personally know someone who identifies as transgender.	29	67	3	43	54	3
14. I have recently (in the past 2 years) encountered a transgender individual in the community.	67	25	8	71	22	7

**Table 4.** **Assessment of UBC and Canadian Medical Students' Basic Knowledge of Transgender Health**

	UBC year 1 (*N*=79)		UBC years 2–4 (*N*=166)		UBC (all years) (*N*=255)		Canada (all years) (*N*=110)	
Question	True (%)	False (%)	True (%)	False (%)	True (%)	False (%)	True (%)	False (%)
15. Gender identity has a biological basis and, once established, it usually remains constant.	35	65	24	76	28	72	41	59
16. A person who identifies as a MALE, but was born FEMALE and has not undergone sex reassignment surgery, can still be considered transgender.	93	7	95	5	95	5	99	1
17. Hormone and/or surgical therapies are appropriate treatment options for transgender patients and should be considered for those patients who request them.	96	4	98	2	97	3	99	1
18. Prescribing cross-gender hormones may result in unacceptable side effects to the degree that they should not be offered.	15	85	24	76	17	83	14	86
19. During MALE-to-FEMALE sex reassignment surgery, the prostate gland is removed.	47	53^a^	24	76^a^	30	70	19	81
20. A 55-year-old MALE-to-FEMALE patient who has been treated with hormones, but has not undergone sex reassignment surgery, should receive regular screening mammograms.	73	27	75	25	74	26	74	26
21. A 55-year-old FEMALE-to-MALE patient who has been treated with hormones, but has not undergone sex reassignment, should receive regular screening pap smears.	87	13	86	14	87	13	93	7

^a^ Significant difference (*p*<0.05).

**Table 5.** **Reported Comfort Level of UBC and Canadian Medical Students with Regard to Their Knowledge of Transgender Health**

	UBC									Canada		
	Year 1 (*N*=79)			Years 2–4 (*N*=166)			All years (*N*=255)			All years (*N*=110)		
	% Agree or strongly agree	% Disagree or strongly disagree	% Neutral	% Agree or strongly agree	% Disagree or strongly disagree	% Neutral	% Agree or strongly agree	% Disagree or strongly disagree	% Neutral	% Agree or strongly agree	% Disagree or strongly disagree	% Neutral
22. A FEMALE patient in your primary care clinic reports feeling like a MALE since adolescence and requests hormonal therapy. I feel sufficiently knowledgeable to assist with this.	0^a^	83	17	9^a^	77	14	6	79	15	7	65	28
23. A MALE patient in your primary care clinic reports feeling like a FEMALE since adolescence and requests hormonal therapy. I feel sufficiently knowledgeable to assist with this.	1^a^	82	17	8^a^	76	15	6	78	16	7	63	30

^a^ Significant difference (*p*<0.05).

Only 29% of UBC students reported personally knowing a transgender individual (Table 3), and only one student at UBC identified as transgender. Similarly, there were no students at other Canadian institutes who identified as transgender and 43% reported personally knowing a transgender individual.

The majority of UBC students (82%) reported being comfortable around transgender individuals (Table 2). Most students also reported that transgender individuals have unique health risks (92%) and that it is important for a physician to know if his/her patient is struggling with gender identity (99%). Similar attitudes were reported by the students who answered the survey at other Canadian medical schools.

Of the students who received the transgender curricula at UBC, 72% reported being introduced to the topic (Table 2), while only 24% felt it was proficiently taught, and 88% wanted to know more. Since the curricular analysis showed that the students at UBC are not introduced to the topic until the end of first year, responses from first-year students were excluded where appropriate. A similar exclusion was not done for the other Canadian institutes as the curricular analysis revealed that the instruction differed greatly among institutions. Comparably, 67% of students felt that they were introduced to the topic, while only 27% of students felt it was proficiently taught, and 88% wanted to know more.

Many students (26% of students from UBC as well as the rest of Canada) failed to recognize that male-to-female patients who are receiving hormones, but have not undergone sex reassignment surgery, do not need screening mammograms (Table 4). Furthermore, when comparing students based on whether or not they were introduced to transgender health in the curriculum, there were very limited differences noted. There was only one question (Question 19) where the responses differed significantly (*p*<0.05); nearly half of first-year students incorrectly answered that the prostate gland is removed in sex reassignment surgery, while only 24% of students in years 2–4 gave this answer.

Very few students (only 6% of UBC students and 7% of students in other schools in Canada) reported feeling sufficiently knowledgeable to address the concerns of a transgender person in a primary care setting (Table 5). When comparing the UBC students who had been introduced to transgender health in the curriculum (in years 2–4) with those who had not (in year 1), there was a minor but significant increase (*p*<0.05) in the number of students who reported feeling comfortable with transgender health. There was no significant difference (*p*>0.05) in the reported comfort level of students at UBC in comparison with students at other Canadian institutes.

## Discussion

This study showed that wide variation exists in the instruction of transgender health among medical schools across Canada. It also showed that the majority of UBC medical students who responded to the survey do not feel sufficiently prepared to address the health concerns of transgender individuals. Despite being interested in transgender health and recognizing that the topic is relevant for their future careers as physicians, these students reported a lack of comfort with providing care to the transgender community.

The surveys from students at other Canadian medical schools largely reflected the findings from UBC; however, the low response rates limit the direct conclusions that can be made from those institutes. The findings are nonetheless provocative and hypothesis generating as the similarities suggest that the gaps identified in transgender health education at UBC may be present in the other Canadian medical schools sampled.

The wide variation in the curricula suggests that among the responding schools, no guidelines are being followed for transgender-related material, and an expectation has not been set for medical students' knowledge and comfort level regarding transgender health. The broad variation is apparent not only with the range of topics introduced but also the quality of coverage. For example, UBC reported spending 2–4 h on transgender health at the end of first year, yet 70% of UBC students did not feel the topic is proficiently taught. This could relate to a lack of consensus regarding which transgender health-related topics are relevant to undergraduate medical student curriculum as well as how much time should be devoted to them. Furthermore, despite being introduced to the topic in the curriculum, a third of UBC students surveyed reported not being introduced to the topic of transgender health at all, suggesting that the material may not have been effectively introduced or reinforced.

In addition, students at UBC are not examined on transgender-related content. Students may prioritize examinable material in their learning and neglect to register material that is not tested by failing to pay attention in class or failing to attend the class altogether. Thus, perhaps there is a need to work toward reevaluating the expectations set for graduating medical students, which in Canada is set by the Medical Council of Canada,^25^ to not only ensure that the material is taught but also that students learn it.

The survey results from UBC also showed that very few students reported feeling comfortable with addressing the health concerns of the transgender community, which is supported by apparent gaps in knowledge regarding basic transgender medical care. This suggests that students are coming into medical school without feeling prepared to care for the transgender community and their medical education is not doing enough to make them feel sufficiently comfortable to do so. Although there appeared to be a small increase in the number of students who reported being comfortable with transgender health in years 2–4 at UBC in comparison with students in year 1, the total number of students who reported being comfortable in those years was still small. The modest increase may be due to the small introduction to transgender health that currently exists in the UBC curricula. If that were the case, this would highlight the potential role that a more thorough and effective introduction to the topic may have on improving students' comfort level with the topic. However, the limited survey responses as well as the many confounding factors that are inherent in a 2–4-year period limit the conclusions that can be drawn from this finding.

The positive attitudes that UBC students reported toward the topic of transgender health and the limited experiences they had with the community seem to provide additional insight into the need for curricular developments in medical school. The nearly unanimous report of positive attitudes toward the topic of transgender health reinforces that students' low comfort level is due to inadequate preparation rather than a lack of interest. It also strengthens the likelihood of benefit from increased exposure to the topic. In addition, many UBC students reported limited experiences with the transgender community. Thus, students are unlikely to be prepared outside the curricular setting to effectively communicate with a transgender person let alone care for them in a healthcare setting. Rather, to ensure students are made to feel comfortable with transgender-related healthcare, effective and thorough preparation in the curricula is needed.

American studies have shown that effective introductions to transgender health can substantially improve the reported comfort level of medical students with regard to the topic.^22,24^ For example, Safer and Pearce^24^ showed that a mandatory and examinable lecture on transgender health, integrated into the second-year endocrinology block, substantially improved the comfort level of medical students. Note that the time spent on the topic was largely comparable with the time currently devoted at UBC, yet a similar improvement in the reported comfort level of UBC students was not seen. This may be due to a number of factors, including the topics covered, the way it was introduced, or the material being examined. However, a more detailed review of curricula is needed to clarify this observation.

Since the transgender community represents a sizeable proportion of our population,^1,7^ medical students are likely to encounter transgender individuals in their future careers, regardless of the specialty pursued. Thus, effective training in transgender medicine should not be restricted to a specific specialty program or optional workshop. Rather, a thorough introduction to transgender health should be integrated into the medical school curricula, exposing all future physicians to the material. This would help ensure that all students are more comfortable with the topic, and at that point, the medical community may be able to better address the needs of transgender individuals in the future.

### Limitations

Although this study provides valuable insight into the current status of transgender health education in Canadian medical schools, several limitations must be considered with both the curriculum data received and the student surveys.

More curricular data about the coverage of transgender health are needed to delineate the findings of this study. Although the data received represent a wide selection of Canadian medical schools, to get the broadest picture of the current state of transgender health instruction nationwide, data from more schools are needed. Furthermore, when asking schools to provide curricular data, program administrators were not asked to explicitly exclude curricula provided in electives. Since electives are inherently experienced by only a small proportion of students, including electives in the reported delivery of transgender-related material would inappropriately inflate the students' exposure to the topic at that institute. Although the questions provided to the program administrators were worded in a manner that implied the inclusion of material that the entire student body would be exposed to, it was not clarified in the acquisition and may have skewed the reports of certain schools.

A future study with more resources available to permit a greater response rate would also be stronger. Although the results of even this small sample were consistent across Canada as well as in line with results seen in similar American studies,^6–8^ additional data, specifically from students outside of British Columbia, would help reinforce the findings of this study. It would also allow for more direct comparisons to be made among specific institutes.

Last, it is important to acknowledge that many biases may confound the interpretation of the student surveys. Self-selection bias may have influenced the sampling of our study, and the results may be more representative of students who have an interest in transgender medicine rather than the entire medical student body as those students would be more inclined to take part in the survey. Reporting bias may have also impacted the accuracy of our results. Since the study asked students to answer some controversial questions on a more sensitive topic, students may have provided answers that did not wholly reflect their beliefs. Rather, they may have provided answers that portrayed themselves in a more positive light. Thus, the student body may not actually have such a positive view of transgender health. However, if consideration is given to these noted confounders, it seems that an even more robust transgender-related curriculum would be required to address negative attitudes in addition to knowledge deficits.

## Conclusion

This is the most comprehensive study to date assessing the topic of transgender health knowledge among medical students in Canada. Despite its limitations, we feel that this study provides valuable insight into the current state of instruction of transgender health and has identified a gap in the education of transgender health in Canadian medical schools.

Although students are interested in transgender health, they are not being adequately prepared in medical school to feel comfortable with the idea of caring for transgender individuals. Future studies should assess the degree of deficit in a more rigorous manner and then the efficacy of an intervention designed to address the deficit.

## Abbreviation Used

UBC: University of British Columbia
